# The decline in mortality due to acute complications of diabetes mellitus in Brazil, 1991–2010

**DOI:** 10.1186/s12889-015-2123-5

**Published:** 2015-08-11

**Authors:** André Klafke, Bruce Bartholow Duncan, Antony Stevens, Roger dos Santos Rosa, Lenildo de Moura, Deborah Malta, Maria Inês Schmidt

**Affiliations:** Post-Graduate Studies Program in Epidemiology, School of Medicine, Federal University of Rio Grande do Sul, Rua Ramiro Barcelos, 2600, sala 419, Porto Alegre, RS CEP 90.035-003 Brazil; Community Health Service, Grupo Hospitalar Conceição, Av. Francisco Trein, 596, Porto Alegre, RS CEP 91.350-200 Brazil; Pan American Health Organization, Setor de Embaixadas Norte, Lote 19, 70800-400, Brasilia, DF CEP 70.312-970 Brazil; Department of Chronic Disease Surveillance and Health Promotion, Secretariate of Health Surveillance, Brazilian Ministry of Health, SAF Sul, Trecho 2, Lote 5/6, Bloco F, Torre 1, Edifício Premium, Térreo, Sala 16, Brasilia, DF CEP 70.070-600 Brazil

## Abstract

**Background:**

Mortality from acute complications of diabetes, a predominantly preventable condition, although controlled in high income countries, remains a major challenge for low/middle income countries. The aim of this study is to describe trends in mortality from acute complications of diabetes between 1991 and 2010 in Brazil, a period during which a national health system was implemented offering broad access to diabetes treatment.

**Methods:**

We obtained the number of deaths listed in the Brazilian Mortality Information System between 1991 and 2010 as due to acute complications of diabetes (ICD-9 250.1, .2, or .3 and ICD-10 E10–14.0 or 1), corrected this number for ill-defined causes of death and incompleteness in mortality reporting, and calculated mortality rates standardized to the world’s population. We describe mortality trends with Joinpoint regressions.

**Results:**

Over this 20 year period, mortality due to the acute complications of diabetes fell 70.9 % (95 % CI 67.2 to 74.5 %), from 8.42 (95 % CI 8.27 to 8.57) deaths per 100000 inhabitants in 1991 to 2.45 (95 % CI 2.38 to 2.52) per 100000 in 2010. The reduction occurred in men and women, in all age groups, and in all regions of Brazil.

**Conclusions:**

Mortality from acute complications of diabetes in Brazil has declined markedly in parallel with the implementation of a national health system providing access to insulin and organization of health care. Further decline is possible and necessary.

## Background

The United Nations and the World Health Organization (WHO) have emphasized the vital importance of prevention and control of the non-communicable diseases (NCDs) in low- and middle-income countries [1], electing to focus actions on four groups of diseases: cardiovascular diseases, cancer, chronic respiratory diseases, and diabetes.

Diabetes mellitus is now estimated to be the fifth underlying cause of mortality globally [2], thus establishing itself as a significant and growing global health issue. Effective approaches to confronting diabetes and the other main NCDs in the midst of the current obesity epidemic have been widely debated, and the importance of strong national health systems has been recently emphasized [3, 4].

Brazil, a middle-income country with 200 million inhabitants, is striving to confront the increased burden of the NCDs. Diabetes is a major part of that burden, as Brazil is listed by the International Diabetes Federation has having the 4th largest number of people with diabetes in the world [5]. Prevalence among adults, most recently estimated by self-report at 6.2 % [6], may well be double that, given incomplete assessment [7], and has grown 25 % between 2006 and 2013 [8].

Much progress has been achieved in the care of those with diabetes over the past two decades. In 1988 a national health system (Sistema Único de Saúde, SUS) which provides free and comprehensive care without user fees, and aims for universal access and reduction of health inequities was implemented [9]. Evaluation of trends during this period in relevant outcomes related to diabetes has rarely been done. In this regard, the prevention of death from acute complications of diabetes is of major importance now to low/middle income countries, since, in principle, these deaths are almost entirely preventable (especially before age 40) [10]. High income countries have made great progress in this regard [11–13], but this task remains a major challenge for low and middle income countries. Furthermore, the relative simplicity of monitoring these deaths provides an important yardstick for gauging the results of actions to prevent and control diabetes.

Within this context, the aim of this study is to evaluate trends in mortality in Brazil due to the acute complications of diabetes (diabetic ketoacidosis, hyperosmolar hyperglycemic state, and hypoglycemia), starting from 1991, shortly after Brazil’s national health system was implemented, and covering a period during which health care for diabetes has been progressively organized. Given that acute complication deaths are relatively more frequent prior to age 40, are more preventable, and contribute importantly to the disease’s burden [14–16], we also investigated trends in deaths prior to age 40.

## Materials and methods

We employed secondary data to characterize national trends in mortality. We downloaded public domain demographic data available from the Brazilian Institute of Geography and Statistics (IBGE) and mortality data reported through Brazil’s Mortality Information System (SIM) for the years 1991 to 2010 [17]. Death from diabetes was defined by one of the following three-character codes of the International Statistical Classification of Diseases and Related Health Problems, 9th Revision (ICD-9): “250” (Diabetes mellitus) or 10th Revision (ICD-10): “E10” (Insulin-dependent diabetes mellitus), “E11” (Non-insulin-dependent diabetes mellitus), “E12” (Malnutrition-related diabetes mellitus), “E13” (Other specified diabetes mellitus), or “E14” (Unspecified diabetes mellitus). A death due to an acute complication of diabetes was defined as any death listing the above codes and also presenting the fourth-character subdivision “.1” (with ketoacidosis), “.2” (with hyperosmolarity), or “.3” (with other coma) in ICD-9 or “.0” (Coma with or without ketoacidosis, hyperosmolar coma, hypoglycemic coma, or hyperglycemic coma NOS) or “.1” (Ketoacidosis or acidosis without mention of coma) in ICD-10. We investigated mortality over the complete age range, and also before 40 years of age. The literature suggests that acute causes are relatively more frequent among diabetes deaths prior to age 40, are more preventable, and given the greater number of years of life lost with each death, contribute importantly to the disease’s burden [14–16].

We used IBGE census (1991, 2000, 2010), IBGE population count (1996), or linear interpolation between the above-listed years to determine population size. The study period was confined to the years 1991 to 2010, as the former year was the first for which data necessary for determining completeness of death reporting were available, and the latter was the last for which mortality data were available.

Brazilian mortality registration has made steady process in completeness of reporting (estimated completeness – 1991: 77.6 %, 2011: 94.2 %): and definition of the cause of death (ill-defined deaths – 1991: 18.2 %, 2010: 6.7 %) over the past two decades [18–20]. In trend analyses, corrections must be made in order to take greater past lack of completeness (deaths not notified) and more frequent ill-defined causes into account. Using an approach similar to that taken in key analyses of the Ministry of Health [21, 22], we corrected reported deaths for both estimated lack of completeness of death registration and for ill-defined causes of death (chapter XVI of ICD-9, “Signs, symptoms and ill-defined conditions” (codes 780–799), and chapter XVIII of the ICD-10, “Symptoms, signs and abnormal clinical and laboratory findings, not elsewhere classified” (codes R00-R99).). The approach to these corrections has evolved over the years. We based correction for lack of completeness for 1991 on the estimated number of deaths furnished by IBGE, and calculated correction factors separately for each gender/age group/region strata. We corrected lack of completeness for the years 2000 to 2010 based on a study of active investigation of undeclared deaths [23] and performed the correction separately for each federative unit. Whenever the number of estimated deaths, after correction, was higher than that reported, we used the ratio of estimated to reported deaths as a correction factor. We applied linear interpolation of the data of 1991 and 2000 to estimate lack of completeness of death registration for the years 1992–1999. We corrected ill-defined causes of death by redistributing these deaths under the assumption that their distribution followed that of reported non-external cause deaths [24, 25].

We calculated the mortality rate for acute complications of diabetes annually. Given relatively small absolute numbers of deaths due to acute complications among those under 40 years of age in the north, center-west, and south regions of Brazil, the means of the annual mortality rate within the periods 1991–1995 and 2006–2010 were used to show trends for these groups. We tested statistical significance in the comparison between the two rates using either the confidence intervals of the rate ratios [26] or the p-value of the z-score for the difference between rates [27], the significance level being set at 0.05.

To facilitate comparisons between countries and across regions within Brazil, we directly standardized mortality rates by age and sex to the world standard population [24, 28]. In these analyses, age was stratified in intervals of 5 years up to 79 (0–4 years, 5–9, 10–14, 15–19, 20–24, 25–29, 30–34, 35–39, 40–44, 45–49, 50–54, 55–59, 60–64, 65–69, 70–74, 75–79), the final strata being 80 years and older. We performed calculations with Stata 10.0. We analyzed trends in mortality from acute complications during the period with the Joinpoint Regression Program, starting with a maximum of 3 joinpoints and using the permutation method to determine the best model fit [29]. Though these numbers represent the universe, and as such do not require confidence intervals, we have included these intervals at times.

The project (number 100056) was approved by the Ethics Committee of Hospital de Clínicas de Porto Alegre. As analyses were based on surveillance databases from the Ministry of Health, no patient consent was necessary.

## Results

Over the 1991–2010 period, 694769 deaths occurred due to diabetes in Brazil, of which 81208 (11.7 %) were due to acute complications. Eighty-eight percent of deaths from acute complications coded by ICD-10 did not specify the type of diabetes (coding E14 - unspecified), and type of diabetes was also not obtainable from the data coded under ICD-9 (less than 30 % of the deaths from acute complications in the period were coded according to ICD-9). With regard to the type of acute complication, from 1991 to 1995 (ICD-9), 11274 (44.6 %) deaths were due to ketoacidosis, 7593 (30.0 %) to hyperosmolarity and 6414 (25.4 %) to other coma; from 1996 onward (ICD-10), 26544 deaths (47.5 %) were due to coma and 29383 (52.5 %) to ketoacidosis or acidosis without coma.

Table 1 shows a progressive reduction in the number of deaths from acute complications in men and women over time. The reduction is more pronounced when examining the age-adjusted mortality rates. In 1991, the mortality rate was 26 % higher in women than in men. The ratio of mortality rates between the sexes decreased progressively over time, being only 5 % higher in women by 2010.

**Table 1:** Mortality rates due to the acute complications of diabetes in Brazil, 1991 to 2010^a^

Year	Men		Women		Female/male ratio^b^
	Deaths	Mortality rate (95 % CI)	Deaths	Mortality rate (95 % CI)	
1991	2070	7.45 (7.25-7.65)	2832	9.36 (9.14-9.58)	1.26
1992	1917	6.50 (6.32-6.69)	2868	9.05 (8.84-9.27)	1.39
1993	2025	6.64 (6.46-6.83)	2845	8.66 (8.45-8.87)	1.30
1994	2117	6.54 (6.36-6.72)	2944	8.07 (7.87-8.27)	1.23
1995	2323	6.96 (6.78-7.15)	3138	8.25 (8.05-8.45)	1.18
1996	1780	5.34 (5.18-5.50)	2576	7.00 (6.82-7.19)	1.31
1997	1724	5.03 (4.87-5.19)	2433	6.19 (6.02-6.36)	1.23
1998	1558	4.14 (4.00-4.28)	2253	5.20 (5.05-5.36)	1.26
1999	1601	4.14 (4.00-4.28)	2260	5.00 (4.85-5.15)	1.21
2000	1667	4.05 (3.91-4.18)	2239	4.56 (4.42-4.70)	1.13
2001	1512	3.61 (3.48-3.74)	2110	4.17 (4.03-4.30)	1.15
2002	1486	3.32 (3.20-3.44)	2136	3.93 (3.80-4.06)	1.18
2003	1484	3.16 (3.04-3.28)	1980	3.47 (3.34-3.59)	1.10
2004	1422	2.87 (2.76-2.98)	2047	3.35 (3.23-3.47)	1.17
2005	1498	2.80 (2.69-2.91)	2124	3.24 (3.13-3.36)	1.16
2006	1417	2.48 (2.37-2.58)	1978	2.75 (2.64-2.85)	1.11
2007	1537	2.54 (2.44-2.65)	2032	2.70 (2.60-2.81)	1.06
2008	1572	2.50 (2.40-2.60)	2037	2.58 (2.48-2.68)	1.03
2009	1479	2.28 (2.18-2.38)	2063	2.52 (2.42-2.62)	1.10
2010	1600	2.39 (2.29-2.49)	2141	2.51 (2.41-2.61)	1.05

CI = Confidence interval

^a^for men and women, /100000 inhabitants, corrected for lack of completeness of death registration and ill-defined causes of death, and standardized to the world population standard

^b^Statistically significant (*P* < 0.001) for all years to 2006 and in 2009, *P* = 0.027 in 2007 and not statistically significant in 2008 and 2010

**Fig. 1:**
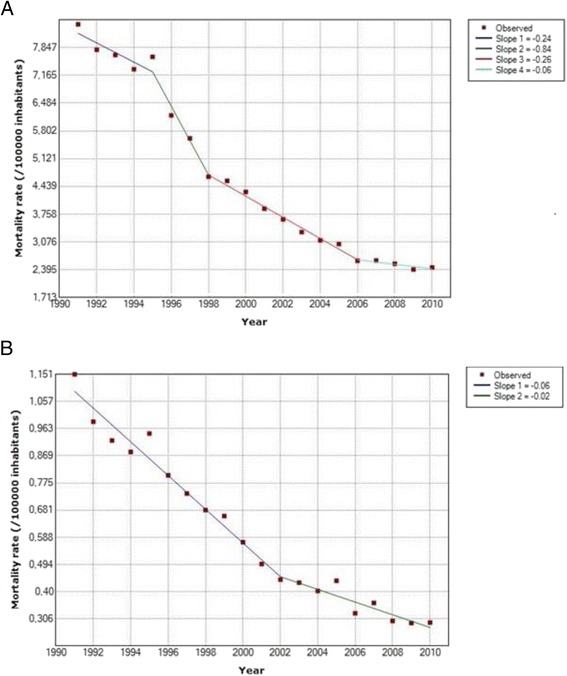
Trends in mortality due to the acute complications of diabetes in Brazil, 1991–2010*. Panel (**a**): All ages. Panel (**b**): Those less than 40 years old. *Corrected for lack of completeness of death registration and ill-defined causes of death, and standardized to the World Population Standard

Figure 1 illustrates rates of mortality due to the acute complications of diabetes in Brazil from 1991 to 2010. Panel a shows a reduction of 70.9 % (95 % CI 67.2 to 74.5 %) in mortality, from 8.42 (95 % CI 8.27 to 8.57) deaths per 100000 inhabitants in 1991 to 2.45 (95 % CI 2.38 to 2.52) deaths per 100000 in 2010. This decline has been less pronounced in recent years.

In 1991, 4902 deaths were due to these complications, accounting for 26.1 % (95 % CI 25.4 to 26.7 %) of all deaths with diabetes as the underlying cause, whereas by 2010, this had declined to 3741 deaths, corresponding to 6.8 % (95 % CI 6.6–7.0 %) of all deaths, a reduction of 73.8 % (95 % CI 69.6 to 78.1 %) in this proportion. Rates of decline produced by Joinpoint regression demonstrate that the greatest rate of decline (a decline of 0.74 deaths/100000 inhabitants/yr) occurred early in the period, from 1995 to 1998.

Panel b of the same figure shows a reduction of 74.5 % (95 % CI 63.0 to 86.1 %) in mortality from these complications in adults under 40 years of age: from 1.15 (95 % CI 1.09 to 1.21) deaths per 100000 in 1991 to 0.29 (95 % CI 0.26 to 0.32) per 100000 in 2010. In 1991, the 646 such deaths that occurred accounted for 53.9 % of all deaths due to diabetes in this age group, whereas by 2010, just 313 acute complication deaths occurred, accounting for only 22.9 % to total deaths due to diabetes, a reduction of 57.5 % (95 % CI 44.0 to 71.0 %) in this proportion. Joinpoint regression again demonstrates a slowing of this decline, the inflexion occurring in 2002.

**Fig. 2:**
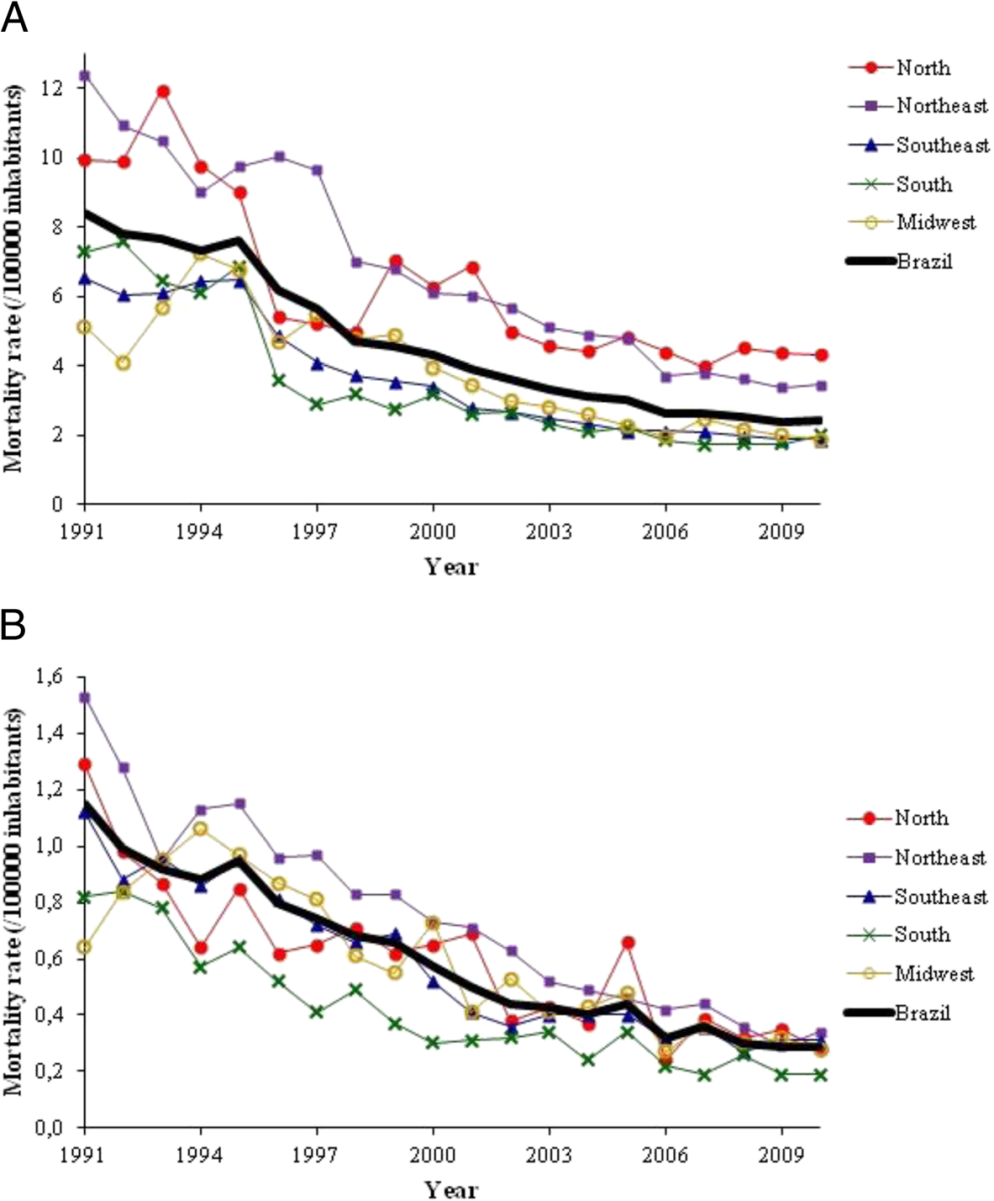
Trends in mortality due to the acute complications of diabetes in Brazil, 1991–2010, by region*. Panel (**a**): All ages. Panel (**b**): Those less than 40 years old. *Corrected for lack of completeness of death registration and ill-defined causes of death, and standardized to the World Population Standard

Panel a of Fig. 2 illustrates that the reduction in mortality occurred in all Brazilian regions (*p* < 0.001, in all regions). The decline was somewhat greater in the Northeast (72.1 %, 95 % CI 66,3-78,0 %), Brazil’s poorest region, than for Brazil as a whole (70.9 %). As can be seen in Panel b of this Figure, the decline in mortality in those under the age of 40 was also observed in all regions of Brazil. Again, the decline was greater in the Northeast (69.5 %, 95 % CI 66.7-72.3 %) than for Brazil overall (67.9 %) considering the average reduction between periods 1991–1995 and 2006–2010.

The decline was observed in all age strata (*p* = 0.039 in the under-fives and *p* < 0.001 in 10 years or more), though not reaching statistical significance in the range of five to nine years (*p* = 0.323), which had the lowest number of deaths (only nine, in both 1991 and 2010).

## Discussion

Mortality due to the acute complications of diabetes in Brazil showed a major decline between 1991 and 2010. Standardized mortality fell approximately 71 % (from 8.4 to 2.4 per 100000), and the fraction of such deaths among those having diabetes as the underlying cause decreased from 26.1 % to 6.8 %. The decline occurred in all regions, in both sexes, and in all age groups, despite the fact that the prevalence of diabetes in Brazil has increased notably over this period [30, 31].

Declines of similar or larger size have been documented over the latter half of the 20th Century in high-income countries, accompanying greater organization of and increased access to health care. In the United States, mortality due to hyperglycemic crises declined from 1.53 to 0.75 deaths per 100000 population over the 30 year period from 1980 to 2009 [11]. When expressed as deaths per estimated number of persons with diabetes, fatality due to acute complications fell by 64 % from 1990 to 2010 [32]. In Japan, a study of patients with diabetes diagnosed before 18 years of age observed a decrease from 421 to 83 deaths from acute complications per 100000 persons with diabetes per year, from the period 1965–1970 to that of 1975–1980 [33]. We know of no studies relating national trends in mortality due to acute diabetes complications in low and middle income countries, home of approximately 80 % of the world’s population affected by diabetes.

Although several factors may have contributed to the marked changes observed in this risk of dying in the abovementioned countries, including improvements in the living conditions of individuals and in health services in general, historical data suggest a critical role for increased access to insulin [34]. Before the availability of insulin, Type 1 diabetes was highly fatal in the first years following diagnosis, as illustrated by the experience of the Joslin Clinic. In the 1897–1914 period, the mortality rate of diabetic children aged 10 at Joslin was 894 per 1000/year [34]. After the introduction of insulin therapy in 1922, this annual mortality fell by more than 80 %, to 61.4 per 1000 during the period 1922–1926. By 1939–1947, following major improvements in availability of insulin and the advent of clinical use of penicillin, mortality declined further, now by more than 90 %, to 3.3 deaths per 1000. And by the period 1950–1961, mortality had declined still further, to 1.0 per 1000 [34]. During the years 1922 to 1929, 14.5 % of deaths were caused by diabetic coma, whereas by 1956–1962 this proportion had decreased to 1.0 % [34].

Thus, we believe that the rapid and marked decline observed in our study resulted, in large part, from similar advances – increased access to insulin, antibiotics and qualified care. Given the time frame of our study, these improvements were largely made possible through the implementation of Brazil’s national health system.

Prior to its implementation, health care was fragmented and access limited, with most public coverage of chronic conditions, other than hypertension, involving hospital care in major urban areas. The expansion of the SUS over the past two decades provided access to an ample range of health care services for the first time to millions of Brazilians with chronic diseases. Currently, an estimated 74 % of the population depends on this public system for their care [35]. The creation of the Community Health Workers Program in 1991 and its expansion into the Family Health Program in 1994 initiated a large primary care network, today called the Family Health Strategy. From that point to the year 2010, over 30000 multidisciplinary teams, consisting of a physician, a nurse, a nurse’s aide and paid community health workers, were organized to provide health care, each covering about 1000 families in a pre-defined territory. Slightly more than 50 % of Brazilians report receiving their care from these teams [30]. An additional fraction, especially in large metropolitan areas, receives free, public ambulatory care in various specialty clinics.

Prior to 1990, access free of charge to insulin was quite limited. In the early 1990s the Ministry of Health produced a National Diabetes Plan, which proposed an equation to calculate the amount of insulin needed per year in Brazil for universal coverage and amplified public purchase of the drug. Throughout the 1990s the restructuring of care for chronic conditions such as hypertension and diabetes at the primary care level progressed, with increasing free public distribution of low cost anti-diabetic medications. In 2001, a major program emphasizing the universal treatment of these conditions at the primary care level, the Plan to Reorganize Care of Hypertension and Diabetes Mellitus [36], was launched. The National Program of Pharmaceutical Provision for Hypertension and Diabetes, created in 2002, and subsequent laws and regulations, have allowed a progressively larger distribution, free of charge, of medicines and medical supplies to the entire population with diabetes in Brazil [37]. Over this period, the SUS also organized the treatment of emergencies, equipping emergency care facilities, providing ambulances, and organizing hotline call systems [38, 39]. In addition to further facilitating access to free medications, more recent efforts have focused on promoting a restructuring of the healthcare system in Brazil, shifting its focus toward the care of chronic conditions [9].

The changes in the rate of the decline within the period studied deserve further discussion. In the early 1990s, when mortality was highest, inadequate access to insulin was likely the major determinant of deaths. Subsequent greater availability of insulin through the SUS during the 1990s may be the main explanation for the sharp reductions in deaths observed. Over time, with the structuring of distribution systems and purchasing processes, and a clearer allocation of responsibilities among different administrative levels of the system, the magnitude of the problem of availability of insulin, other diabetes medications, and supplies diminished greatly. Thus additional, more complex actions, such as the reorganization of care focusing more on the needs of people with chronic conditions, have likely assumed a greater importance in reductions in mortality in more recent years.

Of note, despite this progress, mortality from acute complications of diabetes in Brazil in 2010 was still unacceptably high, 3.3 times greater than what recently reported for the U.S. levels [11]. For those under 40, Brazil’s 2004 mortality level, approximately 0.39 deaths per 100000 population, was similar to that observed between 1968 and 1979 in the state of Washington [40]. Thus, much room exists for additional improvements: strengthening the links which create networks between the relevant parts of the health care system – primary care, emergency care, hospital care, and pharmaceutical delivery, among others; enhancing coordination of care; increasing access to health services, including telephone advice for hyper and hypoglycemia crises [39]; and improving education for physicians and for patients on the identification and management of the acute complications of diabetes [39, 41]. When implemented, these actions need to be tailored so as to take into account remaining regional health inequities as well as the social adversities within which many who have diabetes live.

Brazil is a country of great inequalities, and its national health system has established the goal to diminish those seen in health. At a regional level, the Northeast, the second largest region in terms of population, is considerably poorer than Brazil as a whole. Traditionally residents of this region have had less access to health care. This is reflected in the higher mortality rates seen, in 1991 for this region. As declines, both in terms of relative and perhaps more importantly absolute terms, were greater in the Northeast than overall suggests that progress has been made in reducing health inequalities related to diabetes.

The higher mortality from acute complications of diabetes we found in women has been previously reported by others [42]. A previous Brazilian study also found higher mortality due to all complications of diabetes in women [43]. This differential may merely reflect greater diagnosis of the disease among women [7], which would favor the mention of diabetes on their death certificates. The decrease in the female:male ratio of mortality over time that we found could thus be explained by the decreasing female:male ratio of known diabetes over time.

Given the current stage of both diabetes care and mortality information systems in many low and middle income countries, monitoring of deaths from acute complications of diabetes is a logical indicator to evaluate the quality of medical care offered to those with diabetes in these countries [10]. Use of this mortality statistic has several advantages: comprehensive coverage of the population in countries with compulsory registration of deaths; ability to capture the results of a wide range of actions at all levels of care; simplicity; easy access to data [17]; and the possibility of international standardization of results allowing comparison between countries and over time [44]. Further, our data show that this indicator offers important discriminatory power in settings of greater recent attention to diabetes [44].

Limitations to our analyses deserve mention. Relatively large fractions of deaths were not registered or had ill-defined causes over much of the period studied, particularly during the early years. Although these problems limit the accuracy of our estimates, especially in the initial years studied [25], we believe that the corrective algorithms we implemented have minimized their impact.

## Conclusions

Mortality from acute complications of diabetes in Brazil has declined markedly over the last two decades (1991–2010). Though the decline has occurred across all of Brazil, regional inequalities remain present. The temporal relationship of this decline with the progressive implementation of a national health system offering tax-based universal coverage suggests that the strengths of this health system made this rapid decline possible. Specifically, the goal of providing universal access to health care facilitated diabetes diagnosis and treatment, especially with regard to the availability of insulin and to the ready and appropriate management of acute complications when they present, all of which appears to have contributed to the marked and rapid decline seen.

## Abbreviations

IBGE: Brazilian institute of geography and statistics
ICD-9: International statistical classification of diseases and related health problems, 9th revision
ICD-10: International statistical classification of diseases and related health problems, 10th revision
NCD: Non-communicable disease
SIM: Brazilian mortality information system
SUS: Sistema único de saúde, brazilian national health system
WHO: World health organization
